# Exploring associations between subjective well-being and personality over a time span of 15–18 months: a cohort study of adolescents in Sweden

**DOI:** 10.1186/s40359-021-00673-9

**Published:** 2021-11-05

**Authors:** Regina Winzer, Marjan Vaez, Lene Lindberg, Kimmo Sorjonen

**Affiliations:** 1 Department of Global Public Health, Karolinska Institutet, 17165 Stockholm, Sweden; 2 Department of Living Conditions and Lifestyles, Public Health Agency of Sweden, 17182 Solna, Sweden; 3 Department of Clinical Neuroscience, Karolinska Institutet, 17177 Stockholm, Sweden; 4 Center for Epidemiology and Community Medicine, 10431 Stockholm, Sweden

**Keywords:** Subjective well-being (SWB), Satisfaction with life scale (SWLS), WHO-5 well-being index (WHO-5), Big five inventory (BFI), Positive mental health, Young people

## Abstract

**Introduction:**

Subjective well-being (SWB) is a contributing factor for building resilience and a resource for positive outcomes, e.g. study achievement and work performance. Earlier studies have examined associations between and prospective effects of personality traits on SWB, but few addressed the role that SWB plays in formation of personality over time. The purpose of our study was to examine associations and prospective effects of SWB on personality traits and vice versa in a cohort sample of secondary school students in Sweden who completed self-reported measures of SWB and personality traits at baseline (*N* = 446, 76% females) and at 15–18 month follow-up (*N* = 283, 71% females).

**Methods:**

SWB was defined and measured by the WHO-5 Well-being Index and the Satisfaction with Life Scale. The Big Five Inventory was used to measure personality traits. Autoregressive models were used to analyse associations and potential prospective effects of SWB on personality traits and vice versa.

**Results:**

Low levels of neuroticism and high levels of extraversion, conscientiousness and agreeableness were associated with high levels of SWB at baseline and follow-up. The association between SWB and neuroticism was notably strong. We found high statistically significant rank order stability across the two time points for all measures of personality traits with stability effects, derived from the autoregressive models, ranging from .199 for extraversion to .440 for neuroticism. Stability for SWB was statistically significant across the two time points and ranged from .182 for well-being to .353 for life satisfaction. SWB had a prospective effect on agreeableness only. None of the personality traits had any significant prospective effects on SWB.

**Conclusions:**

The present findings indicate that although correlated, bidirectional prospective effects between personality traits and SWB could not be confirmed. Neuroticism displayed the strongest negative association with adolescents’ SWB. Schools are an appropriate setting to improve well-being, and allocating resources that reduce neuroticism is crucial, including structural interventions, policies for healthy school settings and teaching emotional regulation techniques.

**Supplementary Information:**

The online version contains supplementary material available at 10.1186/s40359-021-00673-9.

## Introduction

Most research on mental health remains rooted in deficit models and focuses on mental ill health, e.g. anxiety, depression and stress rather than on well-being, e.g. emotional and psychological functioning and satisfaction with life. This is also true for research on adolescents’ mental health. Although time trends differ regarding an increase and stability across countries in Europe, Israel and North America [1], a clear, however minor increase in mental health complaints is evident for countries in Northern Europe since the 1980s [2]. In Sweden, young people aged 16–29 have reported the highest levels of deteriorated mental health, i.e. symptoms of anxiety, depression, and stress, over the last decade compared to any other age group [3]. The reasons for these heightened levels of self-reported symptoms are yet unclear, but two possible explanations stated in a literature review have gained the most recognition [4]; the first is a deterioration of the Swedish school system, which has undergone several changes in recent decades including a transformation from a centralised public system to a decentralised system and school choice. In parallel, a new school curriculum was introduced with an increased use of assessments, more strict achievement criteria and high-stakes national tests [5]. These reforms might have led to a decline in school performance and increased degrees of internalised problems among children and adolescents. The second possible explanation concerns the more unstable labour market, which has led to higher demands on education and skills. It is probable that young people feel anxiety and stress about not performing well and about their future position in the labour market. However, alongside higher reported levels of mental health complaints, students’ positive mental health in terms of life satisfaction has not declined, but has instead remained relatively stable since 2001/2002 [6]. This may seem contradictory, but it is possible given that positive and negative mental health may be seen as a dual continuum, whereby an individual can experience well-being despite a status of mental ill health [7, 8]. For example, it is not plausible that a mental health status of diagnosed depression could coexist with high life satisfaction, positive affect and happiness, or low negative affect. On the other hand, symptoms of depression may be transient and certainly can co-exist with stable subjective well-being (SWB).

### Subjective well-being

The concept SWB, referred to in everyday speech as happiness, peace, fulfilment and life-satisfaction [9], is comprised of emotional well-being in terms of a high degree of positive affect and a low degree of negative affect, as well as high levels of satisfaction with one’s life [10]. SWB has been researched for four decades and has been shown to build resilience as well as being beneficial for health, longevity, work performance and supportive social relationships [11]. During adolescence, a period characterized by rapid developmental changes, a high level of SWB is an important tool to navigate life successfully [12]. A representative study from the United States (US) showed that high levels of life satisfaction and positive affect in adolescence and young adulthood correlated significantly with higher levels of income at about age 29 [13]. In college students, high levels of SWB, albeit not the very highest, were associated with study achievement measured by grade point average [14]. In a cross-sectional study among 7th and 8th grade middle school students in the US, those with the highest levels of SWB were also high study achievers [15]. Intervention studies indicate that SWB may be improved through school-based interventions [16].

In addition to measuring individual well-being, SWB is likewise an indicator of societal well-being and varies across nations and according to gross national income [17, 18]. The link between social capital (specifically the dimensions of trust, social interaction, and norms and sanctions) and the individual happiness aspect of SWB has been investigated in 29 European countries [19]. The authors found that all three dimensions matter for happiness, especially social interaction and general social and institutional trust. However, significant differences were found in how social capital interacts with happiness, and in the Nordic countries only trust was associated with happiness. Diener and colleagues have stated that SWB seems to be a necessary, but not sufficient, characteristic of a good life and a good society, and it should be complemented by economic and social indicators [9].

### Associations between personality traits and SWB

Whereas demographic factors such as age and sex are weakly related to SWB, personality traits, together with health and socioeconomic factors, have strong associations according to meta-analyses and reviews [20–23]. The traits extraversion, agreeableness, conscientiousness, neuroticism, and openness to experience are included in the Big Five Model [24–26]. Until the late 1990s, research on personality traits indicated that the mean levels of personality traits change during development in childhood and adolescence, but are principally fixed by age 30 [27]. Later studies rebut these findings and show that changes are possible over the life course and that trait stability continues to increase until after 50 years of age [28], although most changes occur before the age of 40 [29, 30]. Studies on the association between SWB and personality (regardless of study population age) suggest that extraversion is moderately to strongly correlated with positive affect [20, 31–33] and high life satisfaction [21]. Research furthermore shows that neuroticism is associated with negative affect [34–36] and low life satisfaction [21]. In contrast to previous research a recent meta-analysis shows strong associations between conscientiousness and SWB beyond neuroticism and extraversion [23]. Over a period of one year, a test–retest correlation of approximately 0.76 in the measurement of SWB has been observed [37]. Furthermore, over a period of four weeks, a test–retest correlation of approximately 0.88 in the measurement of Big Five personality traits was found [38].

The association between personality and SWB has been mostly studied in adult populations or among young adults [18, 20, 39]. In adolescents, the associations have mainly been assessed in cross-sectional studies without predictive possibilities. In studies on adolescents in corresponding age ranges with our population and conducted in a Western context, neuroticism (low levels), extraversion, and conscientiousness seem to be the strongest factors associated with SWB. This is also apparent in studies on adolescents from Sweden [40], and from the US [41, 42], whereas other studies have only identified significant associations between neuroticism (negatively) and extraversion, and SWB [43, 44] in Swedish and American samples. Nonetheless, a longitudinal study in adolescents on vocational education and training shows another pattern [45]. In this German cohort with a mean age of 18 years, agreeableness and conscientiousness exhibited the strongest associations with life satisfaction at a follow-up measurement of 15 months as well as after three years.

Until recently a common assumption was that personality is relatively stable and causes well-being, but not the reverse [20, 21, 27]. This postulation has been challenged in a large national representative Australian sample of persons aged 15–93 [35]; Soto found that higher levels of extraversion, agreeableness and conscientiousness and emotional stability predicted subsequent levels of SWB. Additionally, high levels of initial well-being predicted agreeableness, conscientiousness, emotional stability, i.e. the counterpart of neuroticism, and introversion, i.e. the counterpart of extraversion, at a later point in time. However, it should be noted that although many of the prospective associations in Soto’s study were significant, due to a large sample (N = 16,367) they were weak, with only one standardized association exceeding 0.1 (0.137). In the present study we intended to investigate if Soto’s findings are maintained when examined in a younger age group, namely adolescents between the ages of 16 and 18. In summary, several scholars before us have longitudinally examined the correlation between personality traits and their effects on SWB over time. Nonetheless, whether SWB may also predict personality has to our knowledge only been investigated and confirmed by Specht [46] and Soto [35] in their studies on predominantly adult populations.

### Purpose of the study

The purpose of this longitudinal study was to analyse associations and prospective effects of SWB on personality traits, and vice versa, over 15–18 month follow up in a cohort sample of girls and boys in secondary schools in Sweden.

## Methods

### Participants and procedure

The sample (*N* = 446, age range 16–18 years, 76% females) at baseline consisted of students in secondary schools in Sweden. Among them, 283 adolescents (71% females) answered the questionnaire at the 15–18-month follow-up. At baseline and follow-up the questionnaires were filled out on a voluntary basis during school lessons and school staff collected the sealed envelopes. The sample was recruited from four secondary schools in middle and south of Sweden. The schools provided academic oriented programs, and the entrance requirements were among the highest in the country. Originally, the recruitment aimed at participation in two school-based intervention studies with controls, with the intention to prevent stress and symptoms of depression. One of the interventions was directed only towards girls, and followed up at 15 months. The other intervention was followed up at 18 months after baseline measurements. All measurements were administered before the COVID-19 pandemic. As the conducted interventions showed no effects on either SWB or personality traits at the follow-up, we merged the intervention and control groups in order to gain a larger sample for the present cohort study. The study has been approved by the Regional Ethical Review Board of the Stockholm Committee (No. 2009/1788–31/3), and informed consent was obtained from the participants.

### Measurements

#### Subjective well-being (SWB)

SWB was defined and measured by two scales: a) the WHO-5 Well-being Index (WHO-5) and b) the Satisfaction with Life Scale (SWLS).

##### WHO-5 well-being index (WHO-5)

Well-being was measured by the World Health Organization Well-being Index (WHO-5). A systematic review of its use in a range of countries and populations, including adolescents, concluded that the WHO-5 tapped into the SWB of the respondents and that the scale is suitable for research on well-being over time [47]. The WHO-5 contains five statements (e.g. *I have felt cheerful and in good spirits*), which are rated on a 6-point Likert scale ranging from 5 = *all of the time* to 0 = *at no time*, and the respondent is asked to reflect on the last two weeks. The raw scores ranging from 0 to 25 are multiplied by 4, where 100 indicates best imaginable well-being. The WHO-5 Well-being Index was translated into Swedish and validated in an adult population sample aged 19 to 64 [48]. The alpha reliabilities of the scale were 0.81 at baseline and 0.67 at follow-up.

##### Satisfaction with life scale (SWLS)

SWB does not only include people’s emotional responses to ongoing life, but also the evaluative factor of life satisfaction [49]. The SWLS has been used in various countries and among different populations, including Swedish adolescents [50]. According to Topp et al. (2015) the WHO-5 is a sufficient instrument for measuring SWB, yet, as it only includes a single item on life satisfaction we also included the SWLS [51] in our measurement of SWB. Another argument to include the SWLS was the finding by Specht et al. that positive changes in life satisfaction were prospectively associated with positive changes in personality traits [46].

In this 5-item inventory, the respondent is asked to rate his or her agreement with statements (e.g. *I am satisfied with my life*) on a 7-point Likert scale ranging from 1 = *strongly disagree* to 7 = *strongly agree*. The items are scored by summing up the raw scores ranging from 5 to 35. The scale has been translated into Swedish and used previously in an adolescent sample [52]. The scale’s alpha reliabilities were 0.82 at baseline and 0.86 at follow-up.

#### Big five inventory (BFI)

The BFI is frequently used to measure personality traits in different countries and populations, including adolescents [53–55]. The personality traits were assessed with the 44-item version by John, Naumann and Soto [26]. The instrument measures extraversion (8 items), agreeableness (9 items), conscientiousness (9 items), neuroticism (8 items), and openness (10 items). All items are rated on a 5-point Likert scale, ranging from 1 = *strongly disagree*, to 5 = *strongly agree*. Scoring instructions were provided by the Berkeley Personality Lab, Institute of Personality & Social Research. The Swedish version of the BFI was used in this study. The instrument has been validated in a Swedish context [56] and in another study involving a sample of adolescents, it was translated from English into Swedish and then back-translated, and no significant discrepancies were found [43]. The scale’s alpha reliabilities at baseline and 15–18 month follow-up were 0.83 and 0.85 for extraversion, 0.73 and 0.69 for agreeableness, 0.81 and 0.81 for conscientiousness, 0.81 and 0.83 for neuroticism, and 0.74 and 0.75 for openness, respectively.

### Statistical analyses

Descriptive statistics means (*M*) and standard deviations (*SD*) were used to outline the distribution of personality traits and SWB at baseline and at 15–18-month follow-up. As SWB and personality traits were measured at only two time points, autoregressive models were used to analyse associations between as well as prospective effects of SWB on the Big Five personality traits and vice-versa. All items of the BFI, the WHO-5, and the SWLS were included in the autoregressive models. As missing values could not be assumed to be missing completely at random (MCAR) listwise deletion was not advisable. Therefore, through multiple imputation, twenty datasets were created in which the missing values were replaced by predicted values. These predicted values varied between the datasets, reflecting uncertainty in such predictions. The parameter values presented below in the autoregressive models are pooled means calculated across the twenty datasets. A sensitivity analysis was conducted by calculating the associations in models employing full information maximum likelihood (FIML) rather than imputations.

**Fig.1:**
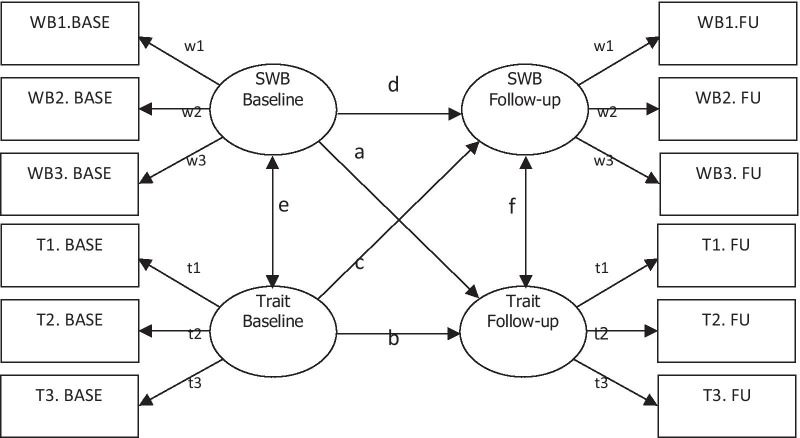
Illustration of the analysed autoregressive models including measures of SWB (measured by the WHO-5 well-being index, and the satisfaction with life scale) and personality traits (measured by the big five inventory) at baseline and at 15–18-month follow-up. The arrows indicate: a = SWB Prospective Effects, i.e. effects of SWB at baseline on Trait at follow-up; b = Trait Stability, i.e. effects of Trait at baseline on Trait at follow-up; c = Trait Prospective Effects, i.e. effects of Trait at baseline on SWB at follow-up; d = SWB Stability, i.e. effects of SWB at baseline on SWB at follow-up; e = Associations between SWB and Trait at baseline; f = Associations between SWB and Trait at follow-up. The error terms for the same item (e.g. T3) were allowed to correlate across the two time points. For measurement invariance, factor loadings (e.g. t3), intercepts, and residuals of the same item were constrained to be equal at the two time points. Parameters b and d indicate rank-order stability and are not affected by possible mean level changes in SWB/personality traits

Following Soto [35], autoregressive models with cross-lagged effects between Big Five personality traits and measures of SWB were fitted to data (Fig. 1). For example, the latent variables extraversion (with eight manifest indicators/items) and SWB (five indicators/items), measured at 15–18 month follow-up, were regressed on extraversion and SWB measured at baseline. The five measures of personality and two measures of SWB resulted in ten analysed models. The error terms of the same item, e.g. item three on the extraversion scale, were allowed to correlate with each other across the two time points. According to Soto, such latent autoregressive models are good at evaluating prospective influences, for example between personality traits and well-being.

In order to achieve measurement invariance, i.e. that the latent variables were comparable measures of the same constructs at the two time points, several constrictions were added to the models. First, the factor loadings of the same item (e.g. item three of extraversion, see parameter t3 in Fig. 1) were constrained to be equal at the two time points. Second, the intercepts of the same item were constrained to be equal at the two time points. Together with the constriction of factor loadings, this means that for the same level of a latent variable, e.g. extraversion, the score on the same manifest indicator, e.g. item three on the extraversion scale, was predicted to be the same at the two time points. Third, the residuals of the same item were constrained to be equal at the two time points. This means that the same amount of variance in a manifest indicator, e.g. item 3 on the extraversion scale, was assumed to be accounted for by variance in the latent variable, in this case extraversion, at the two time points. Without measurement invariance an observed difference between time points could be due to a change in the meaning of the construct rather than in its general level.

Analyses were conducted with R.3.5.0 statistical software [57], employing the packages lavaan [58], semTools [59], and Amelia [60].

## Results

### Personality traits and SWB at baseline and at 15–18-month follow-up

Descriptive statistics of the sample and included measurements at baseline and 15–18-month follow-up are shown in Table 1**.** The cohort sample consisted of *N* = 446, 76% females, at baseline, and *N* = 283, 71% females at follow-up. No significant baseline differences were found between study completers and study dropouts on either the Big Five personality traits or the WHO-5 and SWLS. However, there was a significantly higher attrition rate among girls than among boys. Descriptive statistics for all items and correlations between all individual items as well as between composite scores are presented in the Additional file 1: Table S1.

**Table 1:** Descriptive statistics of the sample and included measurements

	Baseline *N* = 446		15–18-month follow-up *N* = 283	
	Girls *n* = 339 *M (SD)*	Boys *n* = 107 *M (SD)*	Girls *n* = 201 *M (SD)*	Boys *n* = 82 *M (SD)*
Age^a^	16.95 (0.27)	16.97 (0.26)	17.95 (0.28)	17.96 (0.29)
Big five personality traits				
Extraversion	3.55 (0.72)	3.55 (0.69)	3.50 (0.74)	3.59 (0.81)
Agreeableness	3.89 (0.54)	3.72 (0.56)	3.85 (0.56)	3.95 (0.59)
Conscientiousness	3.50 (0.68)	3.41 (0.66)	3.49 (0.70)	3.58 (0.71)
Neuroticism	3.12 (0.70)	2.37 (0.72)	2.90 (0.76)	2.53 (0.61)
Openness	3.79 (0.59)	3.55 (0.63)	3.76 (0.63)	3.68 (0.63)
Subjective well-being				
Well-being (WHO-5)	58.40 (18.60)	66.64 (13.82)	58.65 (18.97)	57.53 (20.86)
Life satisfaction (SWLS)	24.21 (5.64)	24.47 (4.98)	24.63 (6.20)	24.85 (6.26)

^a^Mean age is based on year of birth reported at baseline. Data on month of birth were not available

Standardized parameter values for the ten autoregressive models are presented in Table 2. Associations were obvious between personality traits and SWB when measured at the same time, negative for neuroticism and positive for the others and generally non-significant for openness. There was statistically significant rank order stability across the two time points for the measures of personality traits, with stability effects ranging from 0.199 for extraversion to 0.440 for neuroticism. Similarly, the stability for SWB, i.e. well-being and life satisfaction, was statistically significant across the two time points and ranged from 0.182 for wellbeing to 0.353 for life satisfaction. None of the five trait factors showed any significant prospective effects on well-being and life satisfaction. The only significant cross-lagged effect, 0.203, was shown for well-being on agreeableness. These findings were verified by analyses employing full information likelihood (FIML) estimations rather than imputations (Additional file 2: Table S2).

**Table 2:** Autoregressive standardized effects and correlations with standard error between personality traits and subjective well-being

Subjective well-being	Personality traits				
	Extraversion	Agreeableness	Conscientiousness	Neuroticism	Openness
	β (SE)	β (SE)	β (SE)	β (SE)	β (SE)
WHO-5 well-being index					
Well-being prospective effects (a)	-0.105 (0.078)	0.203 (0.076)**	0.043 (0.073)	0.097 (0.135)	0.055 (0.075)
Trait stability (b)	0.331 (0.075)***	0.292 (0.074)***	0.356 (0.072)***	0.440 (0.133)**	0.352 (0.076)***
Trait prospective effects (c)	0.068 (0.080)	0.006 (0.074)	0.000 (0.075)	-0.150 (0.139)	-0.049 (0.078)
Well-being stability (d)	0.182 (0.084)*	0.283 (0.075)***	0.283 (0.076)***	0.133 (0.142)	0.259 (0.077)***
Associations between well-being and trait at baseline (e)	0.404 (0.079)***	0.192 (0.079)*	0.303 (0.077)***	-0.766 (0.100)***	0.085 (0.079)
Associations between well-being and trait at follow-up (f)	0.467 (0.080)***	0.270 (0.083)**	0.317 (0.079)***	-0.684 (0.097)***	0.190 (0.081)*
Satisfaction with life scale					
Life satisfaction prospective effects (a)	0.139 (0.076)	0.021 (0.069)	0.097 (0.074)	0.066 (0.073)	0.094 (0.069)
Trait stability (b)	0.199 (0.074)**	0.311 (0.073)***	0.322 (0.075)***	0.410 (0.073)***	0.334 (0.072)***
Trait prospective effect (c)	0.051 (0.075)	-0.075 (0.070)	-0.023 (0.072)	-0.036 (0.072)	0.013 (0.071)
Life satisfaction stability (d)	0.305 (0.076)***	0.337 (0.067)***	0.353 (0.073)***	0.323 (0.072)***	0.315 (0.068)***
Associations between life satisfaction and trait at baseline (e)	0.489 (0.075)***	0.280 (0.076)***	0.425 (0.078)***	-0.430 (0.077)***	0.128 (0.075)
Associations between life satisfaction and trait at follow-up (f)	0.426 (0.073)***	0.288 (0.076)***	0.427 (0.079)***	-0.547 (0.082)***	0.125 (0.074)

The letters in parentheses in the first column correspond to parameters in Fig. 1, ***p < .001, **p < .01, *p < .05

β = coefficient standardized in terms of both the predictor and outcome latent variables; SE = standard error of the coefficient

## Discussion

### Main findings and comparisons to other studies

The main finding in this cohort study is that low levels of neuroticism and high levels of, extraversion, conscientiousness and agreeableness are associated with high levels of well-being and life satisfaction in our sample when measured simultaneously at baseline, as well as at 15–18 month follow-up.

The principal contribution of the study was our attempt to test not only if personality traits had prospective effects on SWB, but also if SWB had prospective effects on personality. To our knowledge, the latter test has only been conducted among adults in two different studies [35, 46] but not in a sample of adolescents in secondary education. Our study was not able to replicate Soto’s [35] main findings, namely that SWB may over time predict personality traits in a similar way as personality traits prospectively predict well-being. None of the five personality traits showed any prospective effect on SWB, i.e. well-being measured by the WHO-5 and by the SWLS. However, as stated in the introduction, Soto [35] analysed data from a much larger sample (N = 16, 367) compared to the present study. Consequently, the difference in result could mainly be due to a difference in power rather than a difference in the strength of the associations. The strongest prospective effect of SWB on personality found by Soto [35] was -0.056 and of personality on SWB 0.137, i.e. not very different from the associations in the present study.

Our main finding, the association between the four mentioned personality traits and well-being and life satisfaction in the order as mentioned above., confirms earlier studies in adolescent populations living in high-income countries [40, 42, 43, 61]. For example, in two other Swedish samples of high-school students, albeit cross-sectional studies, neuroticism showed the strongest negative association with SWB [40, 43], followed by conscientiousness and extraversion, respectively. A study involving a sample of Norwegian folk high school students with a mean age of 19 years also found neuroticism (measured as emotional stability) to be more strongly associated with SWB compared to extraversion [61]. In a US sample of high school students, an instrument consistent with the Big Five Model was tested with life satisfaction as outcome. The analyses revealed that 47% of the variance in adolescents’ life satisfaction was related to personality traits and neuroticism (negatively) turned out to be the strongest predictor, -0.72, followed by conscientiousness 0.18, extraversion 0.14, and openness 0.13 [42]. Similar to our study, none of the abovementioned comparative studies showed strong correlations with agreeableness.

The observed test–retest correlation in SWB in the present study was weaker than in a study by Anglim et al., approximately 0.30 versus 0.76 [37]. The difference could, for example, be due to differences in age between the two samples. Further, in our study the test–retest correlation in personality traits was weaker than in a study by Wood et al., approximately 0.35 versus 0.88 [38]. The difference could be due to differences in time between the measurements.

The studies show similarities as well as differences in the results. Potential explanations for these differences include the specific characteristics of the study population, the context where the studies are conducted in, the time frame when the measurement have been undertaken, the study design, and other factors out of control.

### Limitations, strengths, and future research

A lower proportion of boys than girls participated in this study at both baseline and follow-up, which resulted in an uneven group size of boys and girls. The low proportion of boys in our sample limited us from performing an analysis stratified by gender. A larger population sample and hence an increased statistical power might result in more significant effects.

Other limitations are the relatively short follow-up time of 15–18 months, and the absence of further waves of follow-up. A longer time interval between the assessments might have resulted in a personality maturation and higher levels of change over time in personality and aspects of well-being, as shown in other studies [46, 62]. Additional waves of follow-up could have given a more comprehensive picture of the development in stability of traits and SWB.

We and other scholars we refer to, analysed the association and possible prospective effects between personality and SWB. Although SWB is an important factor and measures life satisfaction and hedonic well-being, i.e. brief happiness, it is not sufficient for a complete assessment of positive mental health. To give a more extensive picture of the complexity in well-being, SWB should be complemented by a measurement on eudaimonic well-being, i.e. self- realization and psychological functioning [63]. An adequate measurement may be Carol Ryff’s instrument, which encompasses six constructs of well-being: autonomy, personal growth, self-acceptance, life purpose, mastery, and the ability to have positive relations with others [64]. These constructs have been identified as important factors in being able to cope with challenges in life [65].

Our cohort sample consisted of high-achieving girls and boys in secondary schools with good reputations and the results might therefore be generalisable only to adolescents in corresponding schools. The Swedish school system has been undergoing a structural change since the 1990s, involving the establishment of independent schools and increased possibilities to choose between different schools. In parallel with the rise of independent schools, performance gaps between schools have widened, and increased variations in grades between schools have been shown [66]. The results of our study may therefore be generalisable to those secondary schools with high performance students. A replication of this study with a more representative and diverse study-population would strengthen the results. The inclusion of a measurement on eudaimonic well-being in addition to SWB would also provide a more comprehensive picture of the concept of well-being.

The relative lack of power compared to Soto’s study [35] with a larger sample, has already been mentioned. This increases the risk for missing associations that actually exist, especially if they are weak. On the other hand, as the present study employed more than one test of significance, the risk for type 1 errors, i.e. observing a significant association purely due to chance, is inflated above the nominal 5%. As we analysed ten different models, a conservative Bonferroni correction would require us to use 0.05/10 = 0.005 as the level of significance and this would mean that associations with one or two stars in Table 2 would not be considered significant. However, if we take into consideration that each of these models included six different parameters (not counting the factor loadings), the used significance level would be even lower at 0.05/ (10 × 6) = 0.0008. These corrections would decrease the power of the study, and increase the risk for type 2 errors, even further. Thus, our data should be interpreted with caution. We would suggest a replication of our study with a larger sample to explore if prospective effects of well-being on, for instance, agreeableness would be found.

### Conclusions

The present findings indicate that although correlated, bidirectional prospective effects between personality traits and SWB as found by Soto and colleagues could not be confirmed, and thus may not be a universal phenomenon. However, low power in the present study makes conclusions about lack of effects uncertain. Neuroticism is the trait displaying the strongest negative association with adolescents’ SWB. Allocating resources that reduce neuroticism and elevate well-being in schools is a crucial issue. This could be accomplished through structural interventions and policies that create healthy school settings and school-based health promotion including emotional learning techniques.

## Supplementary Information

**Additional file 1**. Full correlation matrix, correlation composite scores, and item information.

**Additional file 2**.** Table S2** Autoregressive standardized effects and correlations (with SE) between personality traits and subjective well-being (SWB). Analyses conducted with full information maximum likelihood (FIML) estimation.

## Data Availability

The datasets generated and analysed during the current study are not publicly available, as it requires ethical approval to use the datasets according to Swedish legislation. However, the datasets are available from the corresponding author on reasonable request.
