# Supplementary material for: Exploring associations between subjective well-being and personality over a time span of 15–18 months: a cohort study of adolescents in Sweden
Source: BMC Psychol. 2021 Nov 5;9:173. doi: 10.1186/s40359-021-00673-9 (PMC8569843; doi:10.1186/s40359-021-00673-9)
Supplement: Supplementary file 2 — Additional file 2. Table S2 Autoregressive standardized effects and correlations (with SE) between personality traits and subjective well-being (SWB). Analyses conducted with full information maximum likelihood (FIML) estimation. [file 40359_2021_673_MOESM2_ESM.docx]

**Additional File 2, Table S2** Autoregressive standardized effects and correlations (with SE) between personality traits and subjective well-being (SWB). Analyses conducted with full information maximum likelihood (FIML) estimation.

|  | **Extraversion** | **Agreeableness** | **Conscientiousness** | **Neuroticism** | **Openness** |
| --- | --- | --- | --- | --- | --- |
| **Subjective Well-being** | **β (SE)** | **β (SE)** | **β (SE)** | **β (SE)** | **β (SE)** |
| **WHO-5 Well-being Index** |  |  |  |  |  |
| Well-being Prospective Effects (a) | -0.077 (0.087) | 0.213 (0.082)** | 0.049 (0.082) | 0.16 (0.153) | 0.076 (0.082) |
| Trait Stability (b) | 0.316 (0.075)*** | 0.277 (0.072)*** | 0.343 (0.074)*** | 0.478 (0.139)*** | 0.351 (0.07)*** |
| Trait Prospective Effects (c) | 0.05 (0.079) | -0.006 (0.073) | -0.027 (0.077) | -0.093 (0.145) | -0.066 (0.072) |
| Well-being Stability (d) | 0.234 (0.093)* | 0.27 (0.085)*** | 0.271 (0.088)** | 0.159 (0.16) | 0.273 (0.083)*** |
| Concurrent Correlations (e) | 0.414 (0.064)*** | 0.207 (0.066)** | 0.32 (0.066)*** | -0.758 (0.083)*** | 0.093 (0.066) |
| Longitudinal Correlations (f) | 0.454 (0.079)*** | 0.257 (0.081)** | 0.306 (0.079)*** | -0.685 (0.094)*** | 0.171 (0.081)* |
| **Satisfaction with Life Scale** |  |  |  |  |  |
| Life Satisfaction Prospective Effects (a) | 0.149 (0.085) | 0.066 (0.078) | 0.091 (0.08) | 0.074 (0.082) | 0.102 (0.073) |
| Trait Stability (b) | 0.208 (0.08)** | 0.296 (0.076)*** | 0.32 (0.079)*** | 0.389 (0.078)*** | 0.341 (0.071)*** |
| Trait Prospective Effect (c) | 0.017 (0.079) | -0.077 (0.072) | -0.044 (0.078) | -0.032 (0.076) | -0.005 (0.071) |
| Life Satisfaction Stability (d) | 0.325 (0.085)*** | 0.363 (0.074)*** | 0.356 (0.079)*** | 0.319 (0.08)*** | 0.338 (0.07)*** |
| Concurrent Correlations (e) | 0.492 (0.063)*** | 0.282 (0.065)*** | 0.427 (0.066)*** | -0.435 (0.067)*** | 0.111 (0.063) |
| Longitudinal Correlations (f) | 0.417 (0.075)*** | 0.291 (0.08)*** | 0.436 (0.082)*** | -0.562 (0.086)*** | 0.109 (0.075) |
| Note: The letters in parentheses in the first column correspond to parameters in Figure 1, *** p < .001, ** p < .01, * p < .05  β=coefficient standardized in terms of both the predictor and outcome latent variables; SE=standard error of the coefficient | | | | | |
